# Large field-of-view short-wave infrared metalens for scanning fiber endoscopy

**DOI:** 10.1117/1.JBO.28.9.094802

**Published:** 2023-03-09

**Authors:** Ningzhi Xie, Matthew D. Carson, Johannes E. Fröch, Arka Majumdar, Eric J. Seibel, Karl F. Böhringer

**Affiliations:** a University of Washington, Department of Electrical and Computer Engineering, Seattle, Washington, United States; b University of Washington, Department of Mechanical Engineering, Human Photonics Lab, Seattle, Washington, United States; c University of Washington, Department of Physics, Seattle, Washington, United States; d University of Washington, Department of Bioengineering, Seattle, Washington, United States; e University of Washington, Institute for Nano-Engineered Systems, Seattle, Washington, United States

**Keywords:** endoscope, scanning fiber, metalens, large field-of-view, ultrasmall optics, infrared angioscope

## Abstract

**Significance:**

The scanning fiber endoscope (SFE), an ultrasmall optical imaging device with a large field-of-view (FOV) for having a clear forward view into the interior of blood vessels, has great potential in the cardiovascular disease diagnosis and surgery assistance, which is one of the key applications for short-wave infrared biomedical imaging. The state-of-the-art SFE system uses a miniaturized refractive spherical lens doublet for beam projection. A metalens is a promising alternative that can be made much thinner and has fewer off-axis aberrations than its refractive counterpart.

**Aim:**

We demonstrate a transmissive metalens working at 1310 nm for a forward viewing endoscope to achieve a shorter device length and better resolution at large field angles.

**Approach:**

We optimize the metalens of the SFE system using Zemax, fabricate it using e-beam lithography, characterize its optical performances, and compare them with the simulations.

**Results:**

The SFE system has a resolution of ∼140  μm at the center of field (imaging distance 15 mm), an FOV of ∼70  deg, and a depth-of-focus of ∼15  mm, which are comparable with a state-of-the-art refractive lens SFE. The use of the metalens reduces the length of the optical track from 1.2 to 0.86 mm. The resolution of our metalens-based SFE drops by less than a factor of 2 at the edge of the FOV, whereas the refractive lens counterpart has a ∼3 times resolution degradation.

**Conclusions:**

These results show the promise of integrating a metalens into an endoscope for device minimization and optical performance improvement.

## 1 Introduction

In the diagnosis of diseases and surgery in the cardiovascular system, it is essential to have a clear view into the interior of blood vessels. An endoscope with an extremely small diameter (≲1  mm) and short rigid tip length (≲10  mm) can provide a viable solution. To that end, scanning fiber endoscopes (SFEs) have already been demonstrated as one of the smallest forward viewing endoscopes due to their single point scanning based optical sensing mechanism.^1^^,^^2^ Alternative techniques include imaging through a lens on a coherent fiber bundle (CFB) array^3^^,^^4^ or a camera sensor chip-on-tip,^5^ which are difficult to miniaturize while maintaining large field-of-view (FOV) and high resolution. SFEs also feature a large depth-of-focus (DOF) (>10 mm), have a larger FOV, and more pixels than CFB arrays and camera sensors chip-on-tip.^6^

The short-wave infrared (SWIR) wavelength regime is of particular interest for the surgery-assisting endoscopy used inside blood vessels, as they are the shortest wavelength ranges to ensure both reduced scattering and low water absorption. This enables an infrared (IR) endoscope design^7^ to see through blood without requiring any additional clearing. In fact, an SWIR SFE at 1310 nm has already been reported:^8^ here a compound refractive spherical lens is used to project the beam emitted from a scanning single mode fiber to a large range of angles (−35  deg to 35 deg relative to the optical axis). The size of this optical element is one of the major limitation factors for further miniaturization of the rigid tip length of the SFE system. In addition, the spherical lens also suffers from large aberration when the beam is projected to >25  deg off-axis angles.

**Fig. 1:**
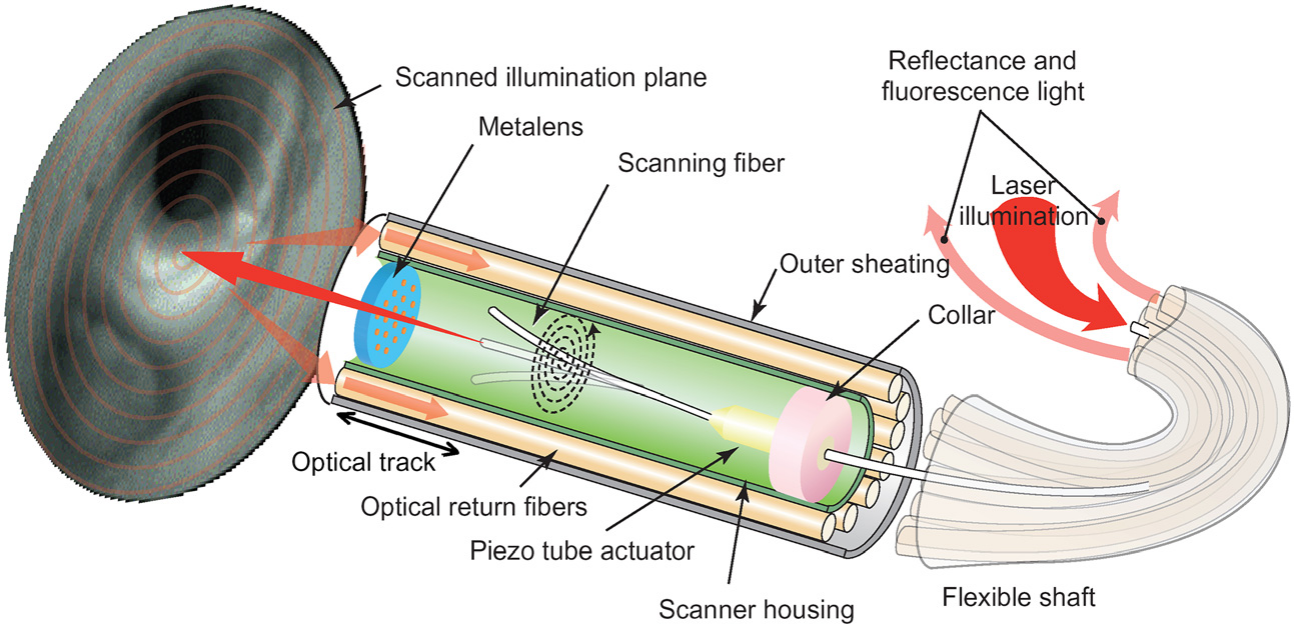
The schematic of a metalens-based SFE system. A suspended single mode fiber is resonantly driven by a piezo electric tube to follow a spiral scanning path, and the beam emitted from the fiber tip at different locations is projected to different angles by a metalens to illuminate the object. As the fiber scans following a spiral path, the beam scans across the whole FOV. The object diffusively reflects the scanning beam, which is collected by the return fibers to form an image of the object.

Metaoptics has recently emerged as a promising alternative to drastically miniaturize optical elements.^9^ Metaoptics consists of quasi-periodic arrays of subwavelength scatterers, each of which can be designed to impart a desired phase, amplitude, polarization, and spectral control of light. The ability to engineer any phase-mask with subwavelength resolution makes metaoptics an excellent candidate to create ultrathin freeform optics.^10^ Metaoptical lenses, commonly known as metalenses, are extremely thin (thickness approaching ∼1  μm for SWIR), and can be fabricated to have very small area. By intelligently designing the phase-mask, metaoptics can correct for aberrations at large off-axis angles, without using multiple optical components.^11^ This ability of metaoptics has already inspired several works on metaoptical endoscopy.^12^^–^^14^ Here, we report an SFE system with a metalens working at 1310 nm to achieve comparable resolution, FOV, and DOF as the state-of-the-art while minimizing the rigid tip-length and reducing the aberration at large off-axis angles. Figure 1 shows the schematic of our SFE system. This imaging system has a full FOV of 70 deg and a DOF of ∼15  mm (in this range, the resolution drops by less than a factor of 2). The replacement of the refractive compound lens with a single metalens reduces the length of the optical track of the SFE from 1.2 to 0.86 mm. The spatial resolution at the center of the field is 140  μm at an illumination distance of 15 mm, which corresponds to an angular resolution of 0.53 deg. This resolution drops by less than a factor of 2 at the edge of the FOV.

## 2 Design and Fabrication of the Metalens

### 2.1 Design of the Metalens

The purpose of the metalens in our SFE system is to project the beam emitted from the tip of the scanning fiber to the illumination plane. Specifically, the beam incident at different positions of the metalens is guided to different angles. The SFE should have a wide range of ∼20  mm imaging distance, wherein the projected beam maintains a small diameter. Therefore, the projected beam should be collimated. This functionality can be realized by a single-layer optical component, which can be approximated by a hyperboloid focusing lens. This lens creates an image of the fiber tip at infinite distance when the tip is at the focal plane of the lens. Therefore, we assume the initial phase profile of the metalens as

$$\begin{array}{c} \Phi_{0}=\frac{f-\sqrt{\rho^{2}+f^{2}}}{\lambda }\cdot 2\pi \end{array}\approx -\frac{\pi }{f\lambda }\rho^{2}\approx -\frac{\pi }{d\lambda }\rho^{2},$$ (1)

where ρ is the radius from the center of the lens, λ is the wavelength (1310 nm), f is the focal length of the lens, and d is the distance between the fiber tip and the metalens, f≈d.

The phase profile of the metalens is then optimized using the ray-tracing simulator *Zemax Optical Studio*. The metalens is represented by a binary2 phase mask whose phase profile is expressed as

$$\Phi =\overset{n}{\underset{k=1}{\sum }}A_{k}\rho^{2k},$$ (2)

where k is the number of order, and $A_{k}$ are the corresponding coefficients that we optimize. To ensure centrosymmetry, only even-order exponents are considered. We set n=3, as the first three terms turn out to be sufficient for our optimization. We use $\Phi_{0}$ (i.e., $A_{1}=-\frac{\pi }{d\lambda },A_{2}=A_{3}=0$) as the starting point for optimization.

**Fig. 2:**
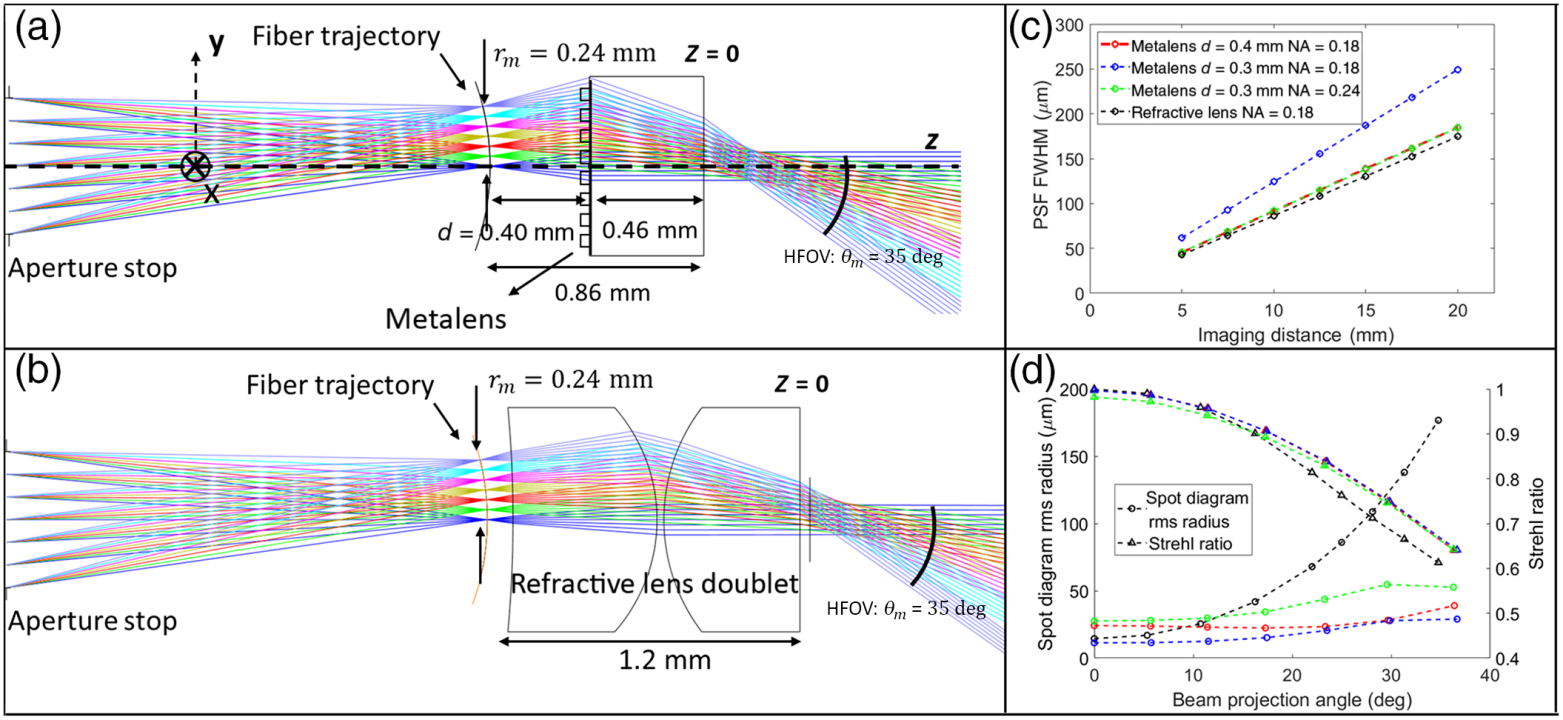
(a) Cross section of the scanning fiber imaging system optimized in Zemax using ray tracing. The object plane simulates the trajectory of the fiber tip during scanning. Each object point position represents one fiber tip position. Seven object points are placed along the y axis with various y values from 0 to $r_{m}=0.24\;\mathrm{mm}$ with an increment of 0.04 mm. An aperture stop is placed ahead of the object surface, which reflects the limited emission solid angle of the beam emitted from the fiber (NA=0.18). The aperture stop size, the object plane curvature, and the distance between the stop and the object are chosen to simulate the scanning behavior of the fiber. A binary2 phase mask is used to model the metalens (aperture diameter: 0.70 mm), which is on a sapphire substrate with the refractive index n=1.75 at the operation wavelength λ=1310  nm. Rays in different colors represent the beam emitted from the fiber tip at different positions and projected to different angles. (b) The design of the state-of-the-art scanning fiber imaging system with the same fiber scanner, but using a refractive spherical lens doublet (Aperture diameter: 0.9 mm). Radius of spherical surface: 0.679 and 0.740 mm. Thickness: 0.579 and 0.547 mm. Material: S-NPH2 and S-LAH58, n=1.87 and 1.85 at 1310 nm), which has the same FOV of 35 deg, but a longer length of 1.2 mm compared with 0.86 mm in panel (a). (c) FWHM of the PSF calculated by Huygens method as a function of the imaging distance, at the center of the field (x=0,y=0). (d) Spot diagram rms radius (from ray tracing) and Strehl ratio (from Huygens PSF) as a function of the beam projection angle for different SFE designs using metalens or refractive lens doublets, at the imaging distance z=20  mm.

Figure 2(a) shows the cross section of our SFE system in the ray-tracing simulation. The trajectory of the fiber tip during scanning is represented by the object plane, whose geometry is predicted by a theoretical model.^15^ The distance between the fiber and lens d, the radius of the object plane $r_{m}$ (which represents the range of motion of the fiber), and the numerical aperture (NA) of the aperture (which is the NA of the fiber) are important design parameters that determine the FOV, DOF, and resolution of the SFE system. d and $r_{m}$ determine the half-FOV $\theta_{m}$ by $\theta_{m}∼\arctan(r_{m}/d)$. A smaller d is desired not only because it reduces the length of the optical track of the SFE system but also requires a smaller fiber scanning range $r_{m}$ to achieve a certain half-FOV. However, the width of the beam w on the metalens (w∼d·NA) also decreases with d, leading to a larger diverging angle ($∼\frac{\lambda }{\pi w}$) of the projected beam, which degrades the DOF and resolution of the SFE system. The fiber we use has an NA of 0.18. We set d=0.4  mm to ensure a relatively short optical track length and acceptable projected beam diverging angle, and $r_{m}=0.24\;\mathrm{mm}$ to get a half FOV of 35 deg. To optimize the SFE performance, the coefficients $A_{1},A_{2}$, and $A_{3}$ are adjusted to minimize the spread of the ray at z=20  mm. Specifically, we choose seven uniformly spaced points along the y-axis in the object plane, and minimize the average radius of the resulting seven spots in the image plane. The point spread function (PSF) is the intensity distribution of the beam projected by the metalens, which determines the resolution of the SFE system. We used the Huygens method in Zemax to calculate the PSF.

Compared with a state-of-the-art SFE system with a refractive spherical lens doublet [Fig. 2(b)], our SFE system with the metalens shows the same FOV of 70 deg, a comparable full-width-at-half-maximum (FWHM) of the PSF at the center of the field [Fig. 2(c)], and a much smaller root-mean-square (rms) radius of the spot diagram as well as a higher Strehl ratio at the projection angle θ>20  deg [Fig. 2(d)]. This means that our metalens can reduce the spherical aberration at large projection angles. The use of the metalens also reduces the optical track length in the SFE from 1.2 to 0.86 mm. This length can be further reduced to ∼0.4  mm using a thinner substrate (∼0.1  mm, which is mechanically strong enough to withstand the fabrication process and retain flatness in SFE setting) and a smaller d∼0.3  mm between the fiber and the lens. As shown in Fig. 2(c), reducing d increases the beam diverging angle, which indicates a degraded resolution and DOF. This can be mitigated by increasing the NA, at the cost of a larger aberration at large beam projection angles.

### 2.2 Meta-Atom Pattern Design

**Fig. 3:**
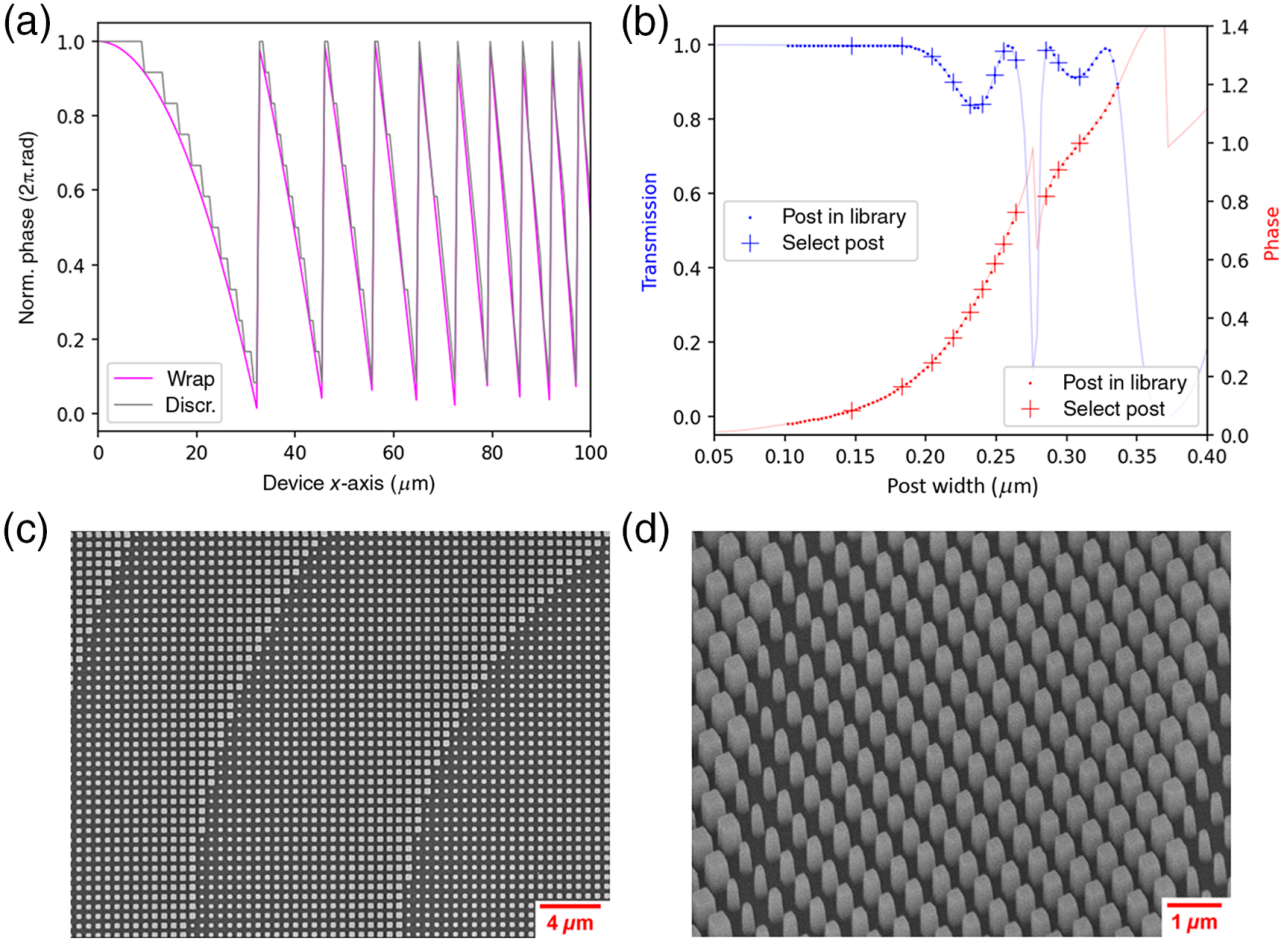
(a) The wrapped and discretized (12 equally spaced levels) phase profile cross section (0<x<100  μm) of the metalens designed in Fig. 2(a). (b) Nanopost phase and transmission response calculated by RCWA, with cSi nanoposts on 460-μm-thick ${\mathrm{Al}}_{2}O_{3}$ substrate at a wavelength of 1310 nm. The height of the nanoposts are h=1000  nm and the periodicity of the nanopost array is p=600  nm. (c) SEM images of the fabricated metalens from the top. (d) SEM image at the oblique angle of 40 deg.

The phase profile of the metalens obtained from the ray-tracing simulation is wrapped and further discretized into 12 levels, as shown in Fig. 3(a). These phase levels are realized by a crystalline silicon (cSi) on sapphire (${\mathrm{Al}}_{2}O_{3}$) metaoptics, in which the subwavelength nanoposts with different sizes act as local scatterer to impose the desired phase response on the incident light. We use rigorous-coupled wave analysis (RCWA)^16^ to theoretically calculate the phase and transmission response versus the scatterer size, as shown in Fig. 3(b). This allows us to pick the right scatterer size at specific locations and thus construct the metalens.

### 2.3 Metalens Fabrication

The metalens is fabricated using an electron beam lithography (EBL) process. A resist (ZEP-520A) on a 1-μm-thick crystalline Si-on-sapphire substrate is patterned by EBL, then a ∼50-nm-thick ${\mathrm{Al}}_{2}O_{3}$ hard mask is created by electron beam-assisted evaporation followed by resist (∼200  nm thick) lift-off in N-methyl-2-pyrrolidone at $90^{{}^{\circ}}C$ overnight. Subsequently, the crystalline-Si layer is etched by a fluorine-based reactive ion process. The scanning electron microscopy (SEM) images of the fabricated metalens are shown in Figs. 3(c) and 3(d).

## 3 Characterization of the Metalens

### 3.1 Experimental Setup

**Fig. 4:**
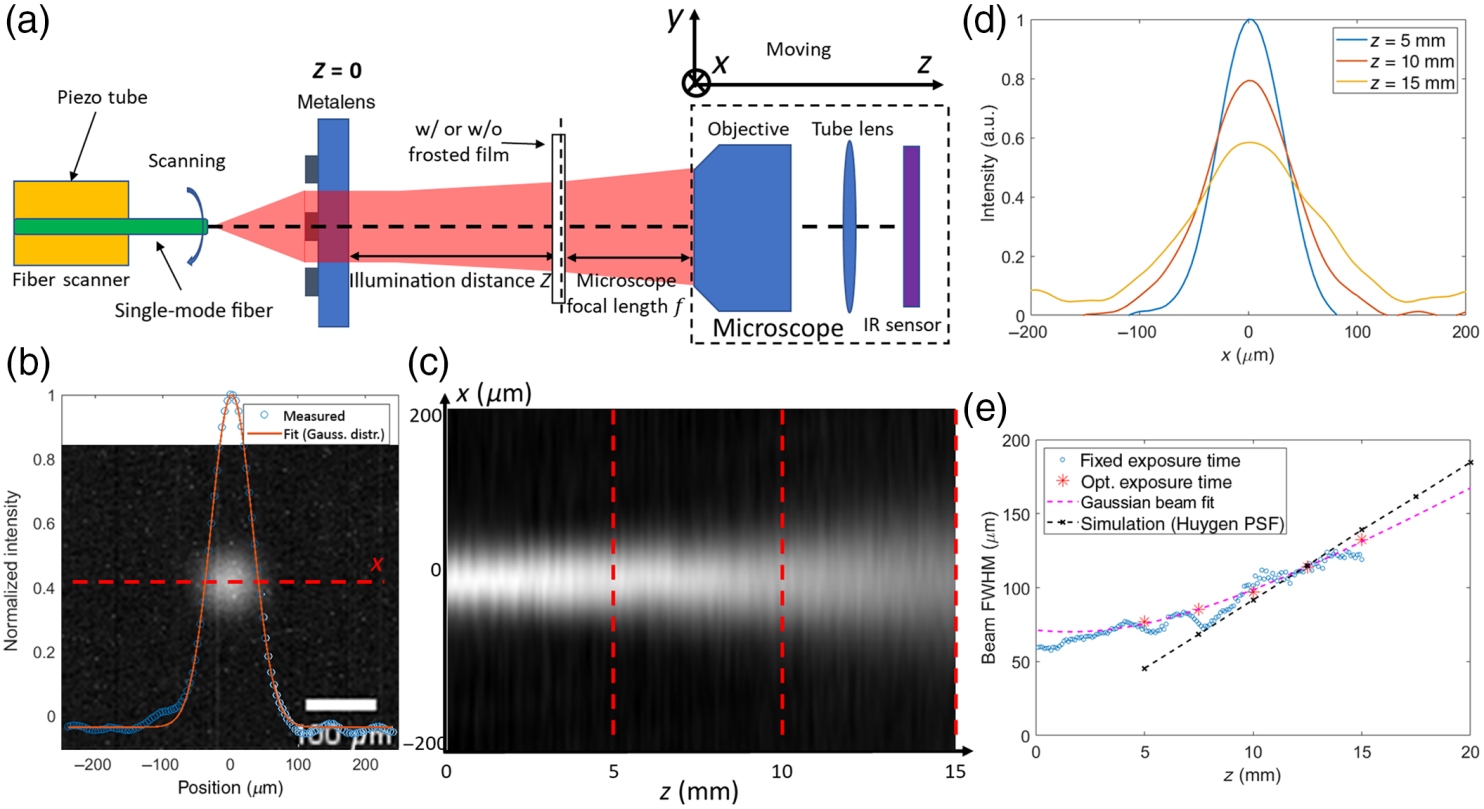
(a) Schematic of the measurement setup for metalens characterization. (b) Direct image of the beam without fiber scanning at the illumination distance z=5  mm. A Gaussian blur (radius = 12  μm) is applied to the image to reduce the noise. After that, the intensity distribution along x-axis (the red dashed line) is subtracted and fitted with a Gaussian distribution function. The measured and fitted intensity distribution is plotted on the image. (c) Longitudinal beam intensity distribution, constructed from a series of images similar to (b) taken at z ranging from 0 to 15 mm with an increment of 0.1 mm. The beam power from the fiber and the exposure time are fixed. (d) Beam intensity distribution along x-axis for various illumination distance z, corresponding to the three red dashed line in panel (c). (e) FWHM of the beam intensity along x-axis versus z at the center of the field. Blue circles correspond to a series of images with a fixed exposure condition. Red stars correspond to images with dynamically optimized exposure conditions to maximize the signal-to-noise ratio. The magenta dashed line is a fit to the red stars, assuming the beam is a perfect Gaussian beam, according to Eq. (3). Fitted Gaussian beam waist width is $w_{0}=59.5\;\mu m$ (corresponding FWHM=70.1  μm), and the waist position is at z=1.58  mm. The black dashed line is the PSF calculated in Zemax according to Huygens method.

The DOF and resolution of our SFE system are determined by the longitudinal (in the x–z plane) and transverse (in the x-y plane, also the illumination plane) intensity distribution of the beam projected by the metalens. Figure 4(a) shows the schematic of the setup for measuring these beam intensity distributions. A customized fiber scanner is built.^8^^,^^15^ The parameters of this fiber scanner are summarized in Table 1. In our SFE, the metalens projects the beam emitted from the tip of the scanning fiber to different angles within a total FOV of 70 deg. A movable IR microscope consisting of an objective (Mitutoyo Plan Apo NIR 10×, f=20  mm, NA=0.26), a tube lens (Thorlabs AC254-075-B-ML, f=75  mm), and an IR sensor (WiDy SenS S320 V-ST) is placed in front of the metalens along the optical axis to characterize the beam on the focal plane of the microscope. This microscope is mounted on a three-dimensional translation stage, which can be moved either along the optical axis (in z-direction) or perpendicular to it (in the x–y plane). By moving the microscope along the z-direction, the beam transverse intensity distribution at different illumination distances z can be measured to construct the longitudinal distribution. By moving the microscope in the x-y plane, the beam intensity distribution at different projection angles can be measured. At beam projection angles >14  deg relative to the optical axis, the objective does not have a large enough NA to capture the light. Therefore, a frosted film is put at the focal plane of the microscope to scatter the beam, and the scattered beam is collected with the microscope.

**Table 1:** Parameters of the customized fiber scanner.

Component	Parameters
Scanner housing	3.8-mm inner diameter (ID), 9.4-mm outer diameter (OD)
Single-mode fiber	0.18 NA, 4.6-μm mode-field diameter, 80-μm cladding diameter
	170-μm coating diameter, 5.5-mm free-standing length
Piezo tube	1.9-mm ID, 2.48-mm OD, 11.6-mm length

### 3.2 Longitudinal Intensity Distribution of the Beam

We first construct the longitudinal intensity distribution (in the x–z plane) of the beam projected by the metalens at 0-deg projection angle (i.e., neutral position of the fiber) by translating the microscope along the optical axis and take images of the beam at different z. Figure 4(b) shows the image of the beam, taken at z=5  mm. The one-dimensional (1D) intensity distribution along the x-axis (the red dashed line) is plotted across the image and further fitted by a Gaussian distribution function. From further measurements of the 1D distributions along the x-axis at different planes in the range z=0 to 15 mm, we construct the longitudinal intensity distribution (in the x–z plane), shown in Fig. 4(c). Importantly, this directly indicates that the beam diverges only slightly over a long distance of 15 mm. Further 1D intensity distributions for different z-distances are plotted in Fig. 4(d), showing how the beam shape is maintained over z=5 to 15 mm while slightly broadening. To further characterize this system, we use an analytical model based on Gaussian beam propagation, summarized in Fig. 4(e), where the FWHM of the 1D intensity distribution as a function of z can be well fitted using

$$\mathrm{FWHM}(z)=w(z)\sqrt{2\ln2},\;w(z)=w_{0}\sqrt{1+[(z-z_{0})/z_{R}{]}^{2}},\;z_{R}=\pi w_{0}^{2}/\lambda .$$ (3)

By fitting, we estimate the beam waist at position $z_{0}=1.58\;\mathrm{mm}$ with waist width $w_{0}=59.5\;\mu m$, which corresponds to an FWHM of 70.1  μm, whereas further along the optical axis at z=15  mm, the beam enlarges to FWHM=135  μm. The FWHM of the 1D intensity distribution of the projected beam increases by less than a factor of 2 when z increases from 0 to 15 mm. Therefore, we define the DOF of our SFE system to be 15 mm. We demonstrate that the beam projected by the metalens has a longitudinal intensity distribution close to a Gaussian beam whose beam waist is at the position of the metalens, which yields the minimal diverging angle for a certain beam width. This ensures a large DOF of 15 mm in our SFE system. The measured beam longitudinal distribution slightly deviates from the simulation results calculated in Zemax ray-tracing simulation using Huygens method [black dashed line in Fig. 4(e)]. We attribute this to the fact that the actual beam from the fiber deviates from our model of a point source.

### 3.3 Scanning Beam FWHM at Various Projection Angles in the Illumination Plane

We characterize the spatial resolution of the SFE system at various angles in the illumination plane by measuring the FWHM of the 1D transverse intensity distribution of the scanning beam. This 1D distribution of the beam can be measured by taking the image of a stable trajectory of the scanning beam with an exposure time exceeding 10× the scanning periodicity of the fiber.

**Fig. 5:**
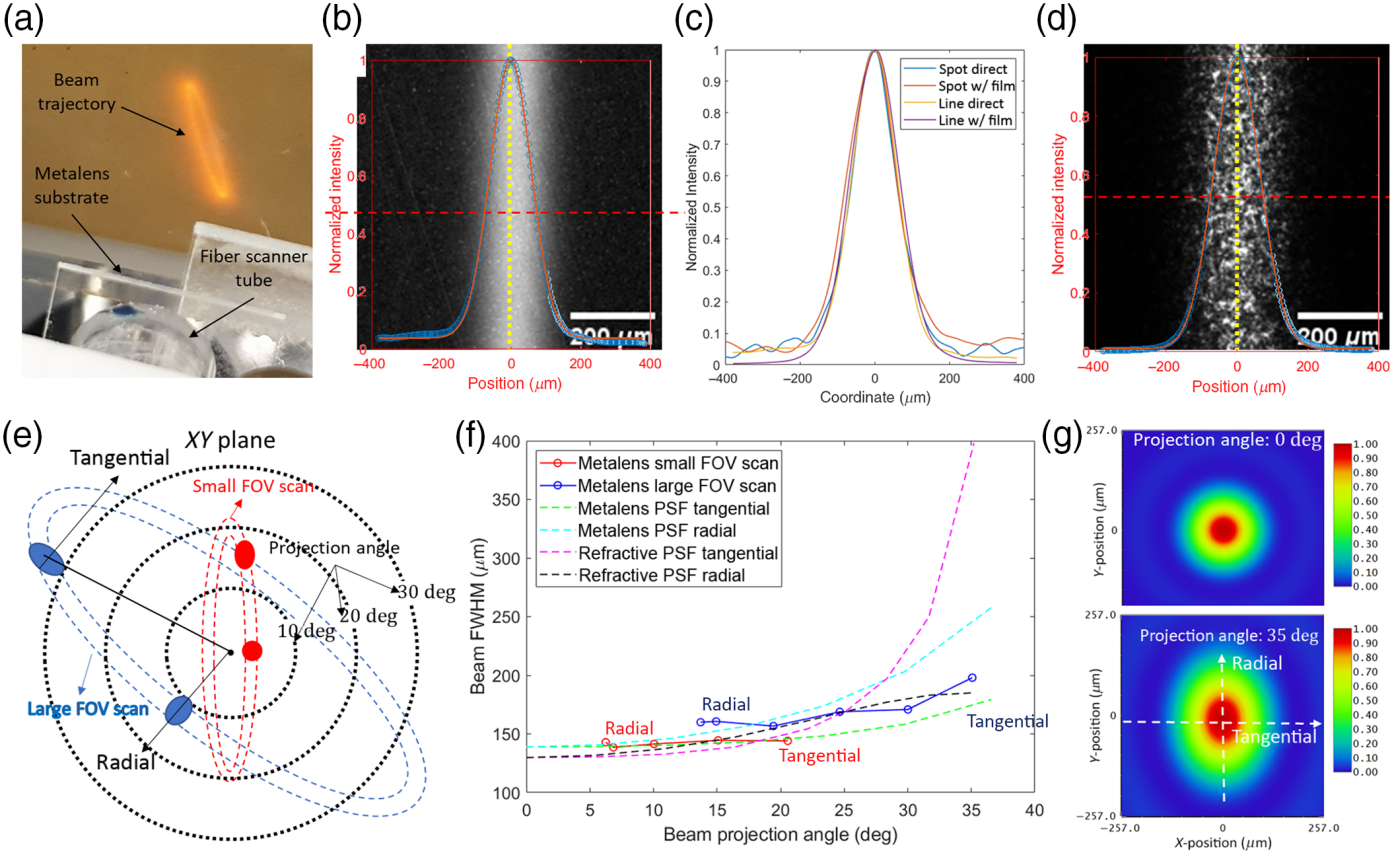
(a) Image of the trajectory of the beam projected by the metalens onto an IR card. The fiber is driven by a piezo tube at the fiber’s mechanical resonance frequency. (b), (d) The center part (6.8-deg beam projection angle) of the beam trajectory with fiber actuation, captured by the IR microscope at the distance z=15  mm, (b) is a direct microscope image, (d) is taken with a thin frost film at the focal plane of the microscope (between the microscope and the metalens). The intensity distribution across the beam trajectory and averaging along trajectory (the yellow dot line) is measured and plotted. Gaussian blur (radius = 12  μm for direct image and 24  μm for image with the paper) is applied to reduce the noise. (c) The normalized intensity distributions across the beam spot (without fiber actuation) and the line (the center part of the scanning trajectory, 6.8-deg beam projection angle), which are measured from the images taken at z=15  mm with and without the frost film. (e) Demonstration of the beam scanning trajectory on the illumination plane. (f) The experimentally measured (solid line) and simulated (dashed line) FWHM of the projected beam vs the projection angle at z=15  mm for the metalens design in Fig. 2(a) and the refractive doublet design in Fig. 2(b). At lower (higher) beam project angle we measure radial (tangential) FWHM. The simulated PSF is calculated using Huygens method in Zemax. (g) PSF of the metalens calculated by Huygens method in Zemax for beam projection angle 0 deg and 35 deg at z=15  mm.

We actuate the fiber scanner at a resonance frequency of 2187 Hz with a stable sinusoidal input signal to scan along an elliptical trajectory. This causes the projected beam to traverse along an elliptical trajectory, as can be seen on an IR card [Fig. 5(a)]. Figure 5(b) shows the center part of the scanning trajectory (6.8-deg projection angle) taken at z=15  mm with an exposure time >8  ms. We measure the intensity distribution across the trajectory (averaging along the trajectory line), which is identical to the 1D intensity distribution of the scanning beam at specific positions. As shown in Fig. 5(c), the 1D intensity distribution of the beam spot without fiber actuation (correspond to 0-deg projection angle) has no observable difference compared with that of the trajectory line of the scanning beam with a small (6.8 deg) projection angle. As the objective lens of our microscope has a small NA of only 0.24, it cannot directly image the beam with projection angles >14  deg. To image the trajectory of the scanning beam at >14  deg, a frost film is placed at the focal plane of the microscope to scatter the light. Figure 5(d) shows the image of beam trajectory on a frost film. The scattering of the frost film causes a slight broadening (∼15  μm) of the FWHM of the intensity distribution. This excess broadening is removed to recover the actual FWHM.

The scanning trajectory at different positions in the x–y plane has different corresponding beam projection angles, as demonstrated in Fig. 5(e). Therefore, to measure the FWHM of the beam at different projection angles, we move the microscope in the x–y plane to take images of different parts of the elliptical scanning trajectory. We scan over a small and large FOV, whose trajectories are high aspect ratio ellipses that cover the projection angle range 6.2 deg to 20.6 deg and 13.7 deg to 35.0 deg [Fig. 5(e)], respectively. We compared the measured FWHM of the scanning beam versus beam projection angle with the simulation results obtained in Zemax (FWHM of the 1D cross section of the PSF calculated by Huygens method) in Fig. 5(f). It is worth noticing that at the projection angle >0  deg, the beam intensity distribution is not centrosymmetric due to the oblique incidence of the beam, which results in beam distortions shown in Fig. 5(g). Specifically, two different distributions along two orthogonal axes arise, which we define as radial and tangential axis. The radial axis connects the beam spot to center (intersection of the optical axis and the x–y plane), and the axis perpendicular to that is defined as the tangential axis. As shown in Fig. 5(e), the intensity distribution across the trajectory is the radial distribution of the scanning beam at the co-vertex of the ellipse (smallest beam projection angle), and is close to the tangential distribution of the beam near the vertex of the ellipse (large beam projection angle). As shown in Fig. 5(f), for both small and large FOV scan, the measured FWHMs at the smallest projection angles of the scans (co-vertex position) are close to the simulated radial FWHMs, whereas at large projection angles of the scans (near to vertex), the measured FWHMs are close to the simulated tangential FWHMs. Therefore, we demonstrate that the measured FWHMs of the scanning beam at various projection angles within the 35-deg half-FOV match well with the simulation. According to the simulation, for our metalens, at the center of the field (0-deg projection angle), the FWHM of the beam is ∼140  μm; the tangential and radial FWHM of the beam increase by 29% and 86%, respectively, at the edge of the FOV (35-deg projection angle). In contrast, when using the state-of-the-art spherical refractive lens doublet [design in Fig. 2(b)], simulations show that the FWHM of the beam is slightly smaller at the center of the field (∼130  μm), but the tangential FWHM of the beam increased by over 187% to 400  μm at the edge of the FOV due to the spherical aberration. The FWHM of the scanning projected beam determines the spatial resolution of our SFE system. Therefore, our metalens SFE system can achieve a comparable resolution of the state-of-the-art SFE at the center of the field, but outperforms the state-of-the-art at the edge of the FOV as it suffers significantly less from resolution degradation (less than a factor of 2).

## 4 Conclusion

We realized a metalens, suitable for integration and miniaturization of an SFE. We demonstrated an SFE system at 1310 nm with large FOV and DOF. Our metalens SFE system can reach an FOV of 70 deg, a DOF of ∼15  mm, and a spatial resolution of ∼140  μm at an illumination distance up to 15 mm at the center of the field, which is comparable with the state-of-the-art. The length of the optics is reduced by 28% (from 1.2 to 0.86 mm), and resolution degradation at the edge of the FOV is reduced to less than a factor of 2. Further device length reduction is possible using a thinner substrate and increasing the NA of the fiber. In addition, the current forward-view design might be modulated to realize side-view imaging. This can be done using a reflective metalens^17^ instead of the transmissive one, and placing it at 45 deg relative to the free-standing fiber. We also note that, while our current implementation employs a single wavelength, using polychromatic metalenses operating in red, green, and blue wavelengths, full color visible SFE can be realized. This could be achieved, for instance by meta-atom engineering, using a scatterer that simultaneously satisfies the phase-profile for multiple wavelengths as demonstrated in the visible range for ∼1-mm aperture and NA of ∼0.7.^18^ Although, these lenses may suffer from a lower focusing efficiency, this could be compensated by increasing the power of the illumination source. We note that in the SFE application, a larger FOV than the reported polychromatic metalenses is needed. However, in SFE, at each beam projection angle, the beam is limited to only a small fraction of the lens, which would reduce the difficulties for large FOV design. Along with meta-atom engineering, other inverse design approaches can be employed, where full-color can be preserved.^13^
